# Cryptochrome Is a Regulator of Synaptic Plasticity in the Visual System of *Drosophila melanogaster*

**DOI:** 10.3389/fnmol.2017.00165

**Published:** 2017-05-30

**Authors:** Milena Damulewicz, Gabriella M. Mazzotta, Elena Sartori, Ezio Rosato, Rodolfo Costa, Elzbieta M. Pyza

**Affiliations:** ^1^Department of Cell Biology and Imaging, Institute of Zoology and Biomedical Research, Faculty of Biology and Earth Sciences, Jagiellonian UniversityKrakow, Poland; ^2^Department of Biology, University of PadovaPadova, Italy; ^3^Department of Genetics, University of Leicester LeicesterUnited Kingdom

**Keywords:** bruchpilot, circadian clock, tetrad synapses, active zone, photoreceptors

## Abstract

*Drosophila* CRYPTOCHROME (CRY) is a blue light sensitive protein with a key role in circadian photoreception. A main feature of CRY is that light promotes an interaction with the circadian protein TIMELESS (TIM) resulting in their ubiquitination and degradation, a mechanism that contributes to the synchronization of the circadian clock to the environment. Moreover, CRY participates in non-circadian functions such as magnetoreception, modulation of neuronal firing, phototransduction and regulation of synaptic plasticity. In the present study we used co-immunoprecipitation, yeast 2 hybrid (Y2H) and *in situ* proximity ligation assay (PLA) to show that CRY can physically associate with the presynaptic protein BRUCHPILOT (BRP) and that CRY-BRP complexes are located mainly in the visual system. Additionally, we present evidence that light-activated CRY may decrease BRP levels in photoreceptor termini in the distal lamina, probably targeting BRP for degradation.

## Introduction

*Drosophila* CRYPTOCHROME (CRY) is a blue light sensitive protein that conveys photic signals to the circadian clock (Rosato et al., 2001; Busza et al., 2004). The strong hypomorphic mutation *cry*_b_ causes aberrant synchronization to light (Emery et al., 1998; Stanewsky et al., 1998), while flies overexpressing *cry* show increased responsiveness to photic stimuli (Ishikawa et al., 1999; Emery et al., 2000). This suggests that CRY modulates the light-dependent regulation of circadian function. The current model of the clock highlights the direct intervention of CRY on the molecular constituents of the circadian system. Evidence has shown that light generates a conformational change in CRY (Ozturk et al., 2011), enabling it to interact with the core circadian protein TIMELESS (TIM, Ceriani et al., 1999). This event triggers the intervention of kinases and E3-ubiquitin ligases. Thus, TIM is phosphorylated, ubiquitinated and degraded by the proteasome (Naidoo et al., 1999; Peschel et al., 2009), explaining its daily oscillations that are in phase with the light-dark (LD) cycle (Hunter-Ensor et al., 1996). Moreover, CRY directly modulates the firing of neurons and influences the circadian system through processes that are independent from the core components of the clock. These involve the redox-sensor function of the voltage-gated K^+^ channel β-subunit (Kvβ) HYPERKINETIC (HK) and additional signaling mechanisms not yet described (Dissel et al., 2014; Fogle et al., 2011, 2015). In line with this findings, CRY accumulates in the projections of neurons where it is expressed (Klarsfeld et al., 2004), it binds to components of the phototransduction pathway in the retinal photoreceptors (Mazzotta et al., 2013) and is involved in magnetoreception (Gegear et al., 2008; Ritz et al., 2010; Fedele et al., 2014a,b; Bazalova et al., 2016). Moreover, CRY has an essential role in circadian plasticity in the lamina: in fact, in *cry*-null (*cry*^0^) mutants the cyclic expression of genes regulating circadian changes in morphology of neurons and synapses is altered (Górska-Andrzejak et al., 2013; Damulewicz et al., 2015).

In our previous work we looked at the rhythmic plasticity of the synapses in the visual system by examining the expression of BRUCHPILOT (BRP) under light/dark (LD 12:12) and constant dark (DD) conditions (Górska-Andrzejak et al., 2013). Using mutants we revealed that the expression of BRP in the distal lamina is under control of both the circadian clock and the light-dark cycle, and that CRY possibly exerts an additional control. In fact, *cry*-null flies showed a reduction in BRP levels at night that was not found in mutants affecting vision (*norp*^A7^) or the clock (*per*^01^, *tim*^01^). BRP is a prominent constituent of the T-bar, and shows homology in its N-terminus to the mammalian active zone proteins ELKS/CAST/ERC (Kittel et al., 2006; Wagh et al., 2006; Fouquet et al., 2009; Hida and Ohtsuka, 2010). In higher Diptera, the T-bar is an electron dense specialization of the presynaptic active zone, which is the site of neurotransmitter release (Wichmann and Sigrist, 2010). BRP is found as two isoforms of 170 kDa and 190 kDa, respectively. They differ in their N-terminal start but their specific functions are not precisely described. Null mutants for *brp* (*brp*^69^) do not produce viable adults but some larvae escape lethality. In those, the model synapse at the larval neuromuscular junction reveals defective active zone membranes, a complete loss of presynaptic specializations and decreased vesicle release (Kittel et al., 2006).

In this study we investigate further the involvement of CRY in the regulation of synaptic plasticity in the visual system, in particular in the lamina, the first optic neuropil.

The results obtained in the present study reveal that CRY forms a complex with the presynaptic scaffolding protein BRP and that it may be involved in the mechanism of BRP degradation in the distal lamina, where the majority of synapses constitute tetrad synapses (Meinertzhagen and O’Neil, 1991).

## Materials and Methods

### Flies Strains

The following strains of *Drosophila melanogaster* were used: Canton S, *w*^1118^ (Bloomington Stock Centre), *cry*^01^—a null mutant of CRY (Dolezelova et al., 2007), *cry*-*GAL4.39* (Picot et al., 2007), *yw;tim*-GAL4 (Emery et al., 1998), *UAS-cry*∆, *UAS-HAcry* (Dissel et al., 2004), *brp*∆^170^, *brp*^∆190^ (Matkovic et al., 2013).

Flies were maintained on a standard cornmeal medium under LD 12:12 regime and at constant 24°C.

### Co-Immunoprecipitation

Flies were collected in liquid N_2_ at specific time points, using red light when sampling the dark phase. The heads were separated from the bodies by vortexing and then were collected using a sieve while still frozen. Fifty heads were used for each extraction. Protein were extracted mechanically (using motor-operated micro-pestles) and by sonication (Hielscher, 60 Hz) in 50 μl of extraction buffer (20 mM Hepes, 100 mM KCl, 2.5 mM EDTA, 5% glycerol, 0.5% Triton X-100, 1 mM DTT, complete protease inhibitors, Roche). The extracts were cleared by centrifugation (1 h at full speed in a microcentrifuge at 4°C) and the supernatants were moved into new 1.5 ml tubes. We precipitated BRP from 50 μl of supernatant using a specific antibody (nc82 [α-BRP], mouse, DSHB) that were bound to Dynabeads magnetic beads (Invitrogen) following the manufacturer’s instructions. Immunoprecipitation reactions were carried out at 4°C overnight. After washing and elution the immuno-complexes were resolved by polyacrylamide gel electrophoresis (PAGE) and Western blot. HACRY was immunoprecipitated as described in Mazzotta et al. (2013).

### Western Blot

Proteins were separated by electrophoresis using commercial polyacrylamide gradient gels 4%–12% (Life Technologies). Proteins were transferred onto a PVDF membrane (Invitrogen) and blocked with 5% powder milk in TBST. The membranes were then incubated overnight with α-CRY (rabbit, 1:500, Dissel et al., 2014), nc82 (mouse, 1:1000, DSHB) or α-HA (mouse, 1:5000, Sigma), as required. TUBULIN (α-TUBULIN, mouse, 1:10,000, Developmental Studies Hybridoma Bank) was used as loading control. For detection we used HRP conjugated secondary antibodies (anti-mouse or anti-rabbit, 1:10,000, Abcam) and a commercial ECL kit (PerkinElmer, Western Lightning Plus-ECL). Protein levels across time points were compared by densitometry (ImageJ). Non parametric Mann-Whitney test was used for data analysis.

### Yeast Two-Hybrid Assays

Two hybrid assays were performed with the LexA/B42 system by Golemis and Brent (1997), using BRP as prey (B42-BRP) and CRY as bait (LexA-CRY) in the yeast strain EGY48 (MAT*α*, *ura3*, *trp1*, *his3*, 3LexA-operator-LEU). The full-length *brp* coding sequence (isoform D) was amplified from cDNA obtained from heads of *w*^1118^ flies with primers pJG_inf_BRP_F (5′-GATGTGCCAGATTATGCCTCTCCCGAATTCGGTACCCATATGATGGGCAGTCA TACTACCGCGAC) and pJG_inf_BRP_R (ACCAAACCTCTGGCGAAGAAGTCCAAAGCTTCTCGAG GGTACCTTAGAAAAAGCTCTTCAAGAAGC) and cloned into the prey vector pJG4-5 using the In-Fusion® HD Cloning Kit (Clontech). The construct was fully sequenced to assess the in-frame insertion of the cDNA and to control for unwanted mutations. The bait construct *pEG202cry* was already available (Rosato et al., 2001). LexA-CRY was challenged with B42-BRP under darkness and under light. As a control LexA-CRY was challenged with B42 only (pJG4-5 empty vector).

Quantification of β-galactosidase activity was performed in liquid culture as in Ausbel (1998) and the experiment was repeated three times. Unpaired *t* test was used for data analysis.

### Immunohistochemistry

Male flies 7 days old were decapitated at *Zeitgeber* Time (ZT, with ZT0 = lights ON, and ZT12 = lights OFF) 1, 4, 13 and 16 under LD 12:12 conditions. Heads were fixed in 4% paraformaldehyde for 4 h, washed twice in PBS, cryoprotected in 12.5% and 25% sucrose, frozen in liquid nitrogen, and then sectioned (20 μm thickness) on a cryostat. The sections were washed in PBS for 30 min and then five times in phosphate buffer with added 0.2% Triton X 100 (PBT). Afterwards, they were incubated in a mix of 5% Normal Goat Serum (NGS) and 0.5% Bovine Serum Albumin (BSA) for 30 min. Mouse nc82 primary antibodies were added to the mix (1:25) and incubated for 48 h at 4°C. The sections were then washed six times in PBT/BSA, blocked in 5% NGS for 45 min and incubated with Cy3 conjugated goat anti-mouse secondary antibodies (Jackson Immuno Research, 1:500), overnight at 4°C. After a series of washes the sections were mounted in Vectashield medium (Vector) and examined with a Zeiss Meta510 Laser Scanning Microscope. Confocal images of the distal lamina were analyzed using ImageJ. The fluorescence intensities of single cartridges were measured as mean gray values. GraphPad Prism software was used for statistics and making graphs. Data were analyzed using one way ANOVA Tukey’s multiple comparisons test.

### Proximity Ligation Assay (PLA)

Canton S and *cry^01^* flies were collected at ZT0. Heads were fixed in 4% paraformaldehyde for 4 h. They were cryoprotected, frozen in liquid nitrogen and then sectioned on a cryostat as 20 μm thick sections. The sections were treated according to the protocol used for immunohistochemistry until the addition of the primary antibodies, α-CRY (1:100) and nc82 (1:25). On the following day the sections were washed and then incubated with the secondary antibodies conjugated to proximity ligation assay (PLA) probes (Duolink). These are short DNA sequences that hybridize to connector oligoes when less than 40 nm apart. The circular structure obtained is then stabilized by ligation and it is amplified by the addition of a rolling circle DNA polymerase. After amplification the newly synthetized DNA is heavily decorated with fluorescent detection probes, making each complex visible under a confocal microscope as a single fluorescent dot.

### Walking Optomotor Response

The walking optomotor response was tested essentially as described by Burnet et al. (1968). Flies were entrained to LD 12:12 conditions. At selected time points (ZT1, ZT4, ZT8, ZT13, ZT16, and ZT20) 7 days old males were placed separately in a T-shaped tube. The longer arm of the T was opaque and located in the center of an arena inside a rotating drum. The internal walls of the drum were painted with alternating black and white vertical stripes, and the apparatus was illuminated from above with a white light (2000 lx). The drum was constantly rotated at 30 rpm. The fly walked out toward the light reaching a choice point where it could turn into the transparent right or left arm of the T-shaped tube. Normal flies are expected to follow the direction of rotation. The test was repeated 10 times for each fly: five times with clockwise and five times with counterclockwise rotation. Each fly was then scored for the number of correct turns taken in the 10 trials. For each time point we analyzed 100 flies for Canton S and *cry* > CRY∆ and 35 flies for the parental (*UAS-cry* and *cry*-Gal4) genotypes. GraphPad Prism software was used for statistics and making graphs. Data were analyzed using one way ANOVA Tukey’s multiple comparisons test.

## Results

### CRY Interacts with the Presynaptic Protein BRP Especially Under Light

**Figure 1:**
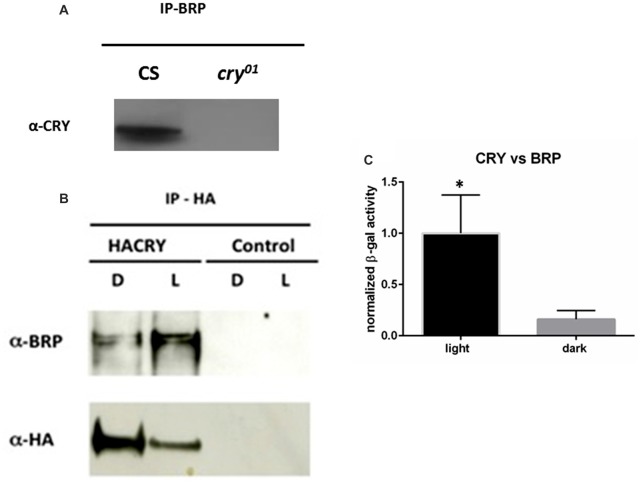
CRYPTOCHROME (CRY) interacts with the presynaptic protein BRUCHPILOT (BRP). **(A)** BRP was precipitated from whole head protein extracts of Canton S and *cry^01^* flies with nc82 antibodies bound onto magnetic beads (Dynabeads). Co-immunoprecipitating (coIP) proteins were resolved by polyacrylamide gel electrophoresis (PAGE), transferred onto membrane by Western blot, and probed with α-CRY primary antibodies. This resulted in a band of *ca*. 60 kDa in the Canton S but not in the *cry*^01^ lane, suggesting a specific CRY-BRP interaction. **(B)** Samples were collected at ZT24 (darkness, D) and at ZT24 + 15 min of light (L) from *tim* > HACRY and *yw;tim*-GAL4 flies, the latter used as a negative control. Whole head protein samples were precipitated with a-HA antibodies and coIP BRP was revealed with nc82 antibodies specifically in flies overexpressing HACRY. BRP is visible as a double band suggesting that both the 170 and the 190 kDa isoforms coIP with CRY. Stronger bands in the L sample suggest that CRY and BRP form a complex more readily under light. **(C)** Full-length CRY (bait) was challenged with full-length BRP (prey) in a yeast 2 hybrid (Y2H) assay with β-galactosidase activity been a measure of interaction. As negative control, full-length CRY was challenged with empty prey vector, and the measured activity was considered as background. The graph reports relative β-galactosidase activity (Miller units) as mean ± SEM of seven independent clones analyzed in triplicate and corrected for background. The asterisk marks a statistically significant difference (*t* test, *p* < 0.0001) between the experiments conducted under darkness and under light.

BRP has a central function in the assembly and maturation of the presynaptic active zone where it interacts with many proteins. Immunoprecipitation of BRP followed by PAGE and silver staining showed co-precipitation of dozens of proteins (Supplementary Figure S1). Some of those were around 65 kDa in size, which is the molecular weight predicted for CRY. Prompted by this observation and by previous work showing a genetic interaction between *cry* and *brp* (Górska-Andrzejak et al., 2013) we explored whether a physical interaction occurs between the two proteins. We precipitated BRP with the nc82 antibody, which targets the C-terminus of the protein hence both the 170 kDa and the 190 kDa isoforms (Matkovic et al., 2013). After PAGE, probing a Western blot with α-CRY antibodies revealed a band in wild type (Canton S) flies but not in *cry*^01^ mutants, suggesting that CRY co-immunoprecipitates with BRP (Figure 1A). Furthermore, we used the mutants *brp*^∆190^ and *brp*^∆170^ expressing only one BRP isoform, the 170 kDa and the 190 kDa type, respectively (Matkovic et al., 2013). Immunoprecipitation with nc82 antibodies followed by Western blot to identify co-precipitating CRY indicated that both isoforms are likely able to form a complex with CRY, although the results were not conclusive due to very week co-immunoprecipitation bands (Supplementary Figure S1). To confirm these results we then increased the expression of CRY using *tim-GAL4* > HACRY flies, namely overexpressing hemagglutinin (HA) tagged CRY in all clock cells. We collected samples at ZT24 (dark) and ZT24 + 15 min light. We precipitated HACRY with α-HA antibodies and probed for BRP on a Western blot. We identified two immuno-positive bands compatible with the 170 kDa and 190 kDa isoforms known for BRP. The interaction was stronger under light conditions (Figure 1B).

Finally, we used a yeast 2 hybrid (Y2H) system to examine whether the physical interaction between BRP and CRY is direct. Full-length CRY was challenged as bait with full-length BRP as prey. We observed a specific increase of β-galactosidase activity and consequent activation of the reporter under light in seven independent clones. Overall these results suggest that light promotes the direct binding between the two proteins (Figure 1C).

### CRY-BRP Complexes Are Formed *In Vivo*

**Figure 2:**
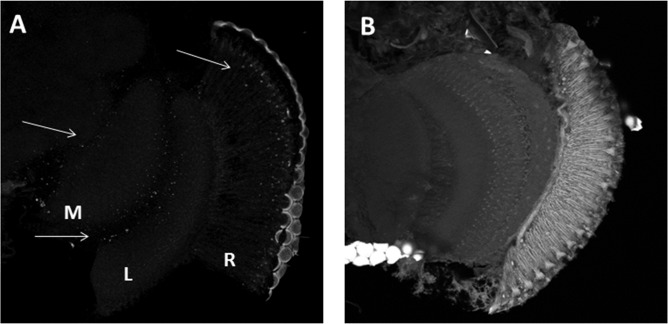
CRY-BRP complexes are formed *in vivo.* CRY-BRP complexes were visualized *in vivo* on 20 μm cryosections of the optic lobe with proximity ligation assay (PLA). **(A)** Canton S and **(B)** *cry*^01^ *Drosophila* brains. Complexes between CRY and BRP are seen as fluorescent dots in the retina (R), the lamina (L) and the medulla (M; arrows in **A**) of Canton S but not *cry*^01^ flies.

We used *in situ* PLA to confirm whether BRP and CRY can form complexes *in vivo*. PLA produces a “dotted” fluorescent signal in regions where two antigens targeted by specific antibodies exist in close physical proximity. Cryostat sections of the optic lobes of Canton S and *cry*^01^ flies where challenged with α-CRY and nc82 antibodies. Confocal analyses revealed fluorescent signals in the retina, in the lamina and in the medulla of Canton S (Figure 2A), but not of *cry*^01^ flies (Figure 2B).

### CRY Affects BRP Degradation in the Lamina

**Figure 3:**
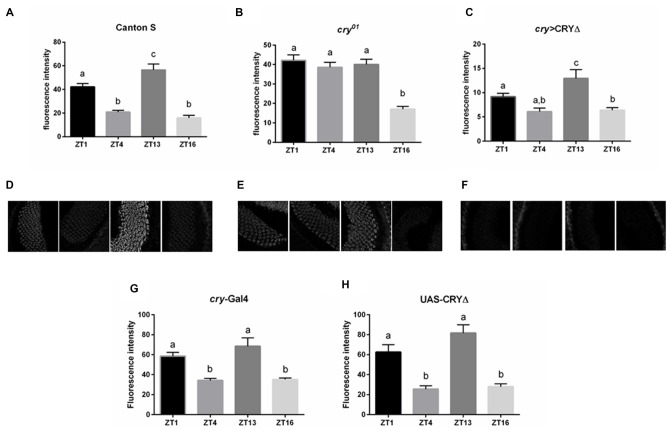
CRY affects BRP degradation in the distal lamina. BRP levels in the distal lamina were measured as immunofluorescence intensity on cryosections of the optic lobe at ZTs 1, 4, 13 and 16. **(A,D)** In Canton S flies the immune-signal was bimodal with one peak at the beginning of the day (ZT1) and one at the beginning of the night (ZT13). **(B,E)** In *cry^01^* mutants, lack of CRY resulted in high BRP immunofluorescence across the whole day (ZTs 1, 4, 13) but a decrease in the middle of the night (ZT16). **(C,F)** In *cry* > CRY∆ overexpressing flies the BRP immune-signal was lower at every time point compared to the other two genotypes. However, a peak was observed at ZT13. **(G,H)** In the parental lines *cry*-Gal4 and UAS-CRY∆ the BRP immune-signal was similar to the one from Canton S, with two peaks at ZT1 and ZT13. Immunofluorescence intensities are reported as Mean ± SD. *N* = 30, three repetitions. Statistical significance was calculated by ANOVA using Tukey’s HSD test. The letters (a,b,c) above the bars represent statistical differences (*p* < 0.05). Namely, the same letter labeling two or more bars indicates that there are not statistical differences between the values. Conversely, two different letters indicate a significant difference between the labeled bars.

**Figure 4:**
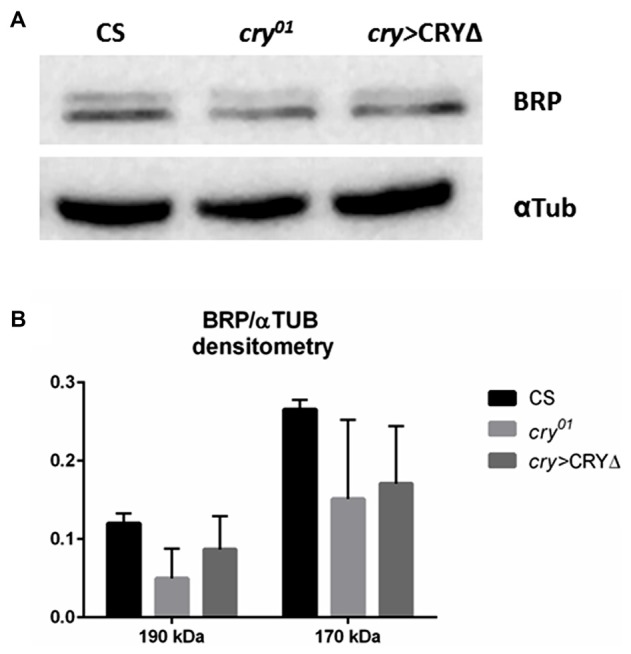
CRY has a complex effect on BRP degradation in whole head protein extracts. BRP levels in whole head protein extracts were determined by Western blot analyses in Canton S, *cry^01^* and *cry* > CRY∆ flies collected at ZT1. **(A)** Representative immunoblot stained with nc82 antibodies showing the 170 kDa and 190 kDa BRP isoforms. Alfa Tubulin (αTub) was used as loading control. **(B)** Densitometry of BRP isoforms normalized to αTub levels. BRP/αTub ratios are reported as Mean ± SD, *N* = 50, three repetitions. A non-parametric Kruskal-Wallis test showed no statistical difference among the strains.

In the lamina the photoreceptor terminals from the retina are arranged in cylindrical modules called cartridges. Beside the photoreceptor terminals R1–R6, they consist of the lamina interneurons and processes of cells projecting from optic neuropils and from the central brain. Within cartridges many synaptic contacts are formed between cells, including tetrad synapses between the photoreceptor terminals R1–R6 and four postsynaptic partners among the following cell types: L1, L2, β-processes of amacrine cells, glial cells or L3 (Prokop and Meinertzhagen, 2006). In the distal lamina BRP expression is rhythmic, showing a light-dependent peak at the beginning of the day (ZT1) and a clock dependent peak at the beginning of the night (ZT13, Górska-Andrzejak et al., 2013 and Figures 3A,D,G,H). The *cry^01^* mutation changes this pattern as BRP levels are high across the whole day and only decrease in the middle of the night (ZT16, Górska-Andrzejak et al., 2013 and Figures 3B,E). This suggests that CRY might be involved in the light-dependent degradation of BRP, possibly providing a functional explanation for the binding between the two proteins in analogy to what it is known for the CRY-TIM interaction. To test this hypothesis we used immunofluorescence to measure BRP levels in the lamina of *cry* > CRY∆ flies. The latter overexpress CRY∆, a C-terminal deletion that results in a constitutively active form of CRY, in all *cry*-positive cells (Rosato et al., 2001; Dissel et al., 2004). *cry* > CRY∆ flies showed maximal immune-signal for BRP at ZT13, but showed no difference between the beginning and the middle of the day (ZT1 and ZT4, see Figures 3C,F). Thus, the pattern of expression is reminiscent of wild type flies under DD (see Figure 2B in Górska-Andrzejak et al., 2013). However, there was an important difference, which is that at each time point the BRP signal was dramatically reduced compared to control (Canton S, *cry*-Gal4, UAS-CRY∆) and *cry*^01^ flies (Figures 3A–H). We also tested by Western blot whether the same pattern of expression might be found for BRP in the whole head. Whole head protein extracts obtained at ZT1 from Canton S, *cry*^01^ and *cry* > CRY∆, were immune-stained with nc82 antibodies. Both *cry*^01^ and CRY∆ flies revealed an overall reduction in the levels of the two BRP isoforms (Figures 4A,B). However, they also showed much greater variability in the expression of BRP than Canton S, as indicated by the large standard deviations reported in Figure 4B. This suggests that CRY affects the regulation of BRP in a more complex and diverse fashion compared to the distal lamina alone.

### CRY∆ Affects the Optomotor Response

**Figure 5:**
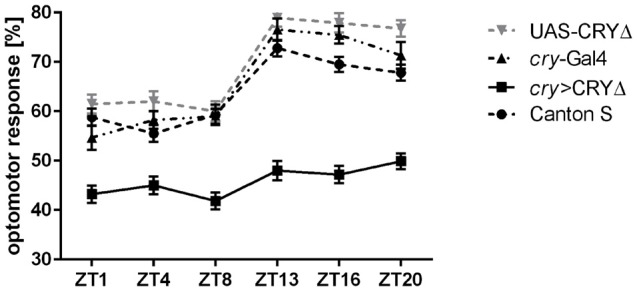
CRY∆ affects the optomotor response. Optomotor walking response of Canton S, *cry*-Gal4, UAS-CRY∆ and *cry* > CRY∆ flies during the day (ZT1, ZT4, ZT8) and during the night (ZT13, ZT16, ZT20). Optomotor responses are reported as Mean ± SE; *N* = 100 for genotypes Canton S and *cry* > CRY∆. *N* = 35 for genotypes *cry*-Gal4 and UAS-CRY∆. Statistical significance was calculated by ANOVA using Tukey’s HSD test. Significant differences. Canton S: ZT1 vs. ZT13 (*p* ≤ 0.0001), ZT1 vs. ZT16 (*p* ≤ 0.001), ZT1 vs. ZT20 (*p* ≤ 0.01), ZT4 vs. ZT13, ZT16, ZT20 (*p* ≤ 0.0001), ZT8 vs. ZT13 (*p* ≤ 0.0001), ZT8 vs. ZT16 (*p* ≤ 0.001), ZT8 vs. ZT20 (*p* ≤ 0.01). *cry*-Gal4: ZT1 vs. ZT13, ZT16, ZT20 (*p* ≤ 0.0001), ZT4 vs. ZT13, ZT16 (*p* ≤ 0.001), ZT4 vs. ZT20 (*p* ≤ 0.0001), ZT8 vs. ZT13, ZT16 (*p* ≤ 0.0001), ZT8 vs. ZT20 (*p* ≤ 0.001). UAS-CRY∆: ZT1 vs. ZT13, ZT16, ZT20 (*p* ≤ 0.0001), ZT4 vs. ZT13, ZT16, ZT20 (*p* ≤ 0.0001), ZT8 vs. ZT13, ZT16, ZT20 (*p* ≤ 0.0001). *cry* > CRY∆: ZT8 vs. ZT20 (*p* ≤ 0.05).

Such a reduced level of BRP in the lamina observed in *cry* > CRY∆ flies should result in measurable effects on vision, which we assessed using the optomotor walking response. Control flies (Canton S, *cry*-Gal4, UAS-CRY∆) showed daily modulation of the optomotor response with about 60% correct choices during the day (ZT1, 4, 8) and about 70%–80% correct choices at night (ZT13, 16, 20). The optomotor response of *cry* > CRY∆ flies was almost flat with significant differences only between ZT8, with about 40% correct choices, and ZT20, scoring about 50% correct choices (Figure 5).

## Discussion

The discovery of CRY was triggered by its ability to modulate the stability of other proteins. The first mutant *cry*^b^ was identified through the loss of rhythmic expression of PER and TIM in peripheral tissues and a dampening of those rhythms in the circadian neurons in the brain (Stanewsky et al., 1998). Subsequently, it emerged that CRY binds to TIM under light (Ceriani et al., 1999) and that such interaction triggers the light-dependent degradation of both proteins (Peschel et al., 2009). This cell-autonomous model of light-induced degradation has dominated the understanding of the circadian function of CRY. However, additional evidence has accumulated showing that CRY must be able to interact with other proteins and to regulate cross-talk among neurons. For instance, antibody staining revealed that CRY accumulates in neuronal projections (Klarsfeld et al., 2004), immune-precipitation of CRY followed by mass spectrometry characterization of co-precipitating proteins has identified components of the phototransduction pathway such as RDGA, INAD and NINAC (Mazzotta et al., 2013), genetic and electrophysiological experiments have associated the voltage-gated Kvβ HK with CRY (Fogle et al., 2015). Moreover, we have shown that CRY affects the expression of BRP, the main constituent of a presynaptic specialization known as the T-bar, in the distal lamina (Górska-Andrzejak et al., 2013).

The lamina is a fantastic model to study synaptic plasticity caused by light input and by the influence of the circadian clock, as synapses and neurons change in size and morphology according to the light regime and endogenous timing (Pyza and Meinertzhagen, 1999; Pyza and Górska-Andrzejak, 2004; Woźnicka et al., 2015).

In the distal lamina BRP levels peak at the beginning of the day (ZT1) and at the beginning of the night (ZT13), which corresponds to an increase in both number and size of tetrad synapses (Górska-Andrzejak et al., 2013; Woźnicka et al., 2015). Interestingly, these changes are paralleled by a rhythm of swelling (morning and evening) and shrinking (middle of the day, middle of the night) of the L1 and L2 monopolar cells (Pyza and Meinertzhagen, 1999).

In the distal lamina of *cry^0^* mutants BRP levels are reduced during the night, whereas during the day BRP expression is constantly high (Górska-Andrzejak et al., 2013). In this study we have used several approaches to show that the interaction between *cry* and *brp* is not only genetic and that the two proteins physically interact. We have pulled-down BRP and identified endogenous CRY as a co-precipitating protein by Western blot (Figure 1A). Moreover, when we pulled-down overexpressed HA-CRY and probed for the endogenous BRP, we observed that both the 170 kDa and the 190 kDa BRP isoforms co-precipitated with HA-CRY (Figure 1B). We interpret this finding as an indirect confirmation of the weak co-immunoprecipitation results obtained with *brp*^∆190^ and *brp*^∆170^ mutants that express only the 170 kDa and the 190 kDa BRP isoform, respectively (Matkovic et al., 2013). In a Y2H assay we verified that CRY and BRP directly bind to each other and that light promotes this interaction (Figure 1C). This result is in agreement with the co-immunoprecipitation experiment presented in Figure 1B, showing that more BRP co-precipitated together with HA-CRY under light conditions than in darkness. We note that although the Y2H experiment suggests a direct interaction between CRY and BRP, additional proteins might be involved *in vivo* to stabilize the complex and/or to initiate a signaling cascade.

Using PLA with anti-BRP and anti-CRY antibodies we discovered PLA-positive signal in the retina, in the lamina and in the medulla of Canton S but not of *cry^01^* flies, showing that the interaction between CRY and BRP occurs *in vivo* and *in situ* (Figure 2). The pattern of fluorescence is in agreement with the known distribution of CRY (BRP is found in all photoreceptors and neurons) but it was surprisingly sparse. We interpret this result as a consequence of the complexity of the technique. One possibility is that the PLA-positive signal is limited to those areas where CRY and BRP are expressed at the highest level. It is unlikely that within tissue sections the primary and the secondary antibodies will always bind with the right steric arrangement to allow the best interaction between the antibodies-bound PLA probes and the connectors. Another possibility is that 20 μm sections are quite difficult to penetrate by enzymes such as ligase and rolling circle DNA polymerase. Hence signal may be prevalent in areas that are more exposed and/or have a “looser” structure. Although we did not investigate these possibilities, it is likely that both aspects played a role in determining the tissue distribution of the PLA-signals we revealed.

To test whether BRP may be targeted for degradation following its interaction with CRY, we turned to CRY∆, a C-terminal deletion of CRY. We have previously shown in a Y2H assay that CRY∆ binds to TIM independently from light, and that the overexpression of CRY∆ in flies results in phenotypes suggesting that this form of the protein does not require activation by light (Rosato et al., 2001; Dissel et al., 2004). For instance, in CRY∆ flies TIM is expressed at lower levels than in wild type, which agrees with the idea of a constitutive interaction with an active CRY. Moreover, CRY∆ does not accumulate or marginally so; this is also expected, considering that the interaction between TIM and CRY drives the degradation of both proteins (Peschel et al., 2009). Thus we hypothesized that *cry* > CRY∆ flies would show reduced immunostaining for BRP at each time point. Indeed, that is what we observed in the distal lamina in comparison to both Canton S and *cry*^01^ flies (Figure 3). This suggests that BRP, like TIM, is targeted for degradation following the formation of a complex with CRY. Although the overall BRP immune-signal was reduced, we could still detect a significant oscillation in BRP levels. At its peak at ZT13, the BRP signal was about twice the size than for the other time points (Figure 3C). Interestingly, this expression profile mimics the temporal distribution of BRP immune-signal in wild type flies maintained under constant darkness (compare the expression profiles of BRP in Figure 3C to Figure 2B of Górska-Andrzejak et al., 2013). This is in spite of the fact that constitutive active CRY, which would arguably simulate constant light, might be expected to produce a non-rhythmic phenotype. When we measured BRP levels in whole heads we observed the reduction of BRP in both *cry*^01^ and *cry* > CRY∆ in comparing with Canton S; however, these differences were not statistically significant due to a remarkable variability among experiments (Figure 4B). Our interpretation is that although CRY does have an effect on the expression of BRP in general, the mechanisms are complex and possibly tissue/brain area specific. We also note that CRY is not uniformly expressed across the brain or the head. Thus, we might have detected a mixture of direct and indirect effects, and arguably the latter might amplify noise. Thus, we conclude that CRY affects BRP expression in the distal lamina, likely regulating its stability. In addition, we propose that the lamina is a particularly attractive model to investigate the mode of action of CRY.

Finally we reasoned that such a reduction in BRP expression in the distal lamina of *cry* > CRY∆ flies (Figure 3), should give rise to behavioral phenotypes. The optomotor response measures the ability to detect and respond to a moving environment. When the environment moves it generates an apparent self-motion to which a spectator responds with movement to stabilize their apparent course. The optomotor response depends to a large extent on the time of day, with best performances observed between the end of the day and the middle of the night and it reflects the presence of a functional circadian clock in the photoreceptor system (Barth et al., 2010; Mazzotta et al., 2013; Mazzotta and Costa, 2016). In order to study the motion vision of flies, we analyzed their optomotor walking response. As previously reported (Mazzotta et al., 2013) Canton S flies performed better at night (ZT13, 16, 20) than during the day (ZT1, 4, 8) with 70% and 60% correct choices, respectively (Figure 5).

Light adaptation in the retina depends on horizontal migration of screening pigment granules towards the rhabdomeres (Nilsson and Ro, 1994). In *Musca domestica* screening pigment granules migrate also vertically in photoreceptors, with maximal accumulation in the proximal part of the lamina at the end of day and higher pigment number in the distal lamina at the end of night. This pattern is clock-dependent (Pyza and Meinertzhagen, 1997). Because the absence of screening pigment causes loss of visual acuity (Burnet et al., 1968), the daily changes in optomotor walking response may be correlated with the pattern of pigment granules migration. However, BRP levels peak at ZT1 and ZT13 in the distal lamina of Canton S (Figure 3A), and we would have expected a similar behavioral outcome at these two time points. Thus, the optomotor response did not reflect the daily differences in number and size of tetrad synapses of which BRP levels in the distal lamina are a proxy. Nevertheless, we could see significant differences between the performances of control and of *cry* > CRY∆ flies (Figure 5). The latter genotype showed 40%–50% of correct choices during the whole day, which is the value expected by chance alone. Thus, these flies either could not detect the movement of the stripes or they were unable to process the information, or may be both. Again we did not see a correlation between the expression profile of BRP and the optomotor response. In an earlier study on the housefly, we found that motor stimulation is more effective than visual stimulation in eliciting morphological changes in the lamina. Thus the lack of correlation between BRP levels and optomotor response is not surprising (Kula and Pyza, 2007). This behavioral assay tests the functioning of the visual system from photoreceptors to higher order motion vision processing neurons in the lobula plate, a region in the optic lobes that is the final destination of visual information (Heisenberg and Wolf, 1984). Our results are in agreement with the previous observation where flies in which CRY lacked its C-terminus tail (*cry*^M^, Busza et al., 2004) showed a reduced performance in the optomotor response (Mazzotta et al., 2013). In addition, flies with *brp* expression silenced in the visual system (*gmr* > *brpRNAi*) showed changes in the electroretinogram (ERG; Wagh et al., 2006), which measures extracellular activity of photoreceptors and interneurons in response to light (Heisenberg, 1971). Lack of BRP in the photoreceptor terminals causes severe defects in synaptic transmission which is visualized in ERG, since light-induced depolarization of photoreceptors is normal but ON/OFF transients originating from the interneurons are absent (Wagh et al., 2006). We speculate that the presence of a constitutively active form of CRY (due to the absence of its regulatory C-terminus) likely impacts on the organization of the visual system beyond the photoreceptors and the lamina and may have similar effect on the retina functionality as *brp* silencing. This consideration calls for additional studies to dissect the role of CRY in the visual system of *Drosophila*.

In conclusion, we have identified a physical interaction between CRY and BRP, the main constituent of the presynaptic active zone T bar. We have confirmed this interaction using different techniques such as immune-precipitation, Y2H and PLA. Our data suggest that CRY and BRP can interact directly especially under light, but they do not preclude that CRY and BRP might be part of a larger complex *in vivo*. We have evidence that CRY regulates the stability of BRP in the distal lamina, possibly mirroring what is known for the CRY-TIM interaction. However, the regulation of BRP in the head is more complex, since not all synapses peak in number at the same time during day and night. Finally, we have presented data showing that the functionality of the visual system is compromised in *cry* > CRY∆ flies, probably affecting higher order processing neurons. This suggests that the role exerted by CRY on the development and physiology of the visual system is greater than currently appreciated, with important consequences for the interpretation of the effects of CRY on the light entrainment of the circadian clock.

## Author Contributions

MD, GMM and ES carried out experiments. MD prepared figures. MD, GMM and ER, RC and EMP discussed results and reviewed the manuscript. MD and ER wrote the main manuscript text.

## Conflict of Interest Statement

The authors declare that the research was conducted in the absence of any commercial or financial relationships that could be construed as a potential conflict of interest.

## References

[B1] Ausbel F. M. (1998). Current Protocols in Molecular Biology. New York, NY: John Wiley & Sons.

[B2] Bazalova O. Kvicalova M. Valkova T. Slaby P. Bartos P. Netusil R.. (2016). Cryptochrome 2 mediates directional magnetoreception in cockroaches. Proc. Natl. Acad. Sci. U S A 113, 1660–1665. 10.1073/pnas.1518622113 26811445 PMC4760799

[B100] Barth M. Schultze M. Schuster C.M. Strauss R. (2010). Circadian plasticity in photoreceptor cells controls visual coding efficiency in *Drosophila melanogaster*. PLoS One 5:e9217 10.1371/journal.pone.0009217 20169158 PMC2821403

[B3] Burnet B. Connolly K. Beck J. (1968). Phenogenetic studies on visual acuity in *Drosophila melanogaster*. J. Insect Physiol. 14, 855–860. 10.1016/0022-1910(68)90196-0

[B4] Busza A. Emery-Le M. Rosbash M. Emery P. (2004). Roles of the two *Drosophila* CRYPTOCHROME structural domains in circadian photoreception. Science 304, 1503–1506. 10.1126/science.1096973 15178801

[B5] Ceriani M. F. Darlington T. K. Staknis D. Más P. Petti A. A. Weitz C. J.. (1999). Light-dependent sequestration of TIMELESS by CRYPTOCHROME. Science 285, 553–556. 10.1126/science.285.5427.553 10417378

[B6] Damulewicz M. Loboda A. Bukowska-Strakova K. Jozkowicz A. Dulak J. Pyza E. (2015). Clock and clock-controlled genes are differently expressed in the retina, lamina and in selected cells of the visual system of *Drosophila melanogaster*. Front. Cell. Neurosci. 9:353. 10.3389/fncel.2015.00353 26441524 PMC4569741

[B7] Dissel S. Codd V. Fedic R. Garner K. J. Costa R. Kyriacou C. P.. (2004). A constitutively active cryptochrome in *Drosophila melanogaster*. Nat. Neurosci. 7, 834–840. 10.1038/nn1285 15258584

[B8] Dissel S. Hansen C. N. Özkaya Ö Hemsley M. Kyriacou C. P. Rosato E. (2014). The logic of circadian organization in *Drosophila*. Curr. Biol. 24, 2257–2266. 10.1016/j.cub.2014.08.023 25220056 PMC4188814

[B9] Dolezelova E. Dolezel D. Hall J. C. (2007). Rhythm defects caused by newly engineered null mutations in *Drosophila’s* cryptochrome gene. Genetics 177, 329–345. 10.1534/genetics.107.076513 17720919 PMC2013679

[B10] Emery P. So W. V. Kaneko M. Hall J. C. Rosbash M. (1998). CRY, a *Drosophila* clock and light-regulated cryptochrome, is a major contributor to circadian rhythm resetting and photosensitivity. Cell 95, 669–679. 10.1016/s0092-8674(00)81637-2 9845369

[B11] Emery P. Stanewsky R. Hall J. C. Rosbash M. (2000). A unique circadian-rhythm photoreceptor. Nature 404, 456–457. 10.1038/35006558 10761904

[B12] Fedele G. Edwards M. D. Bhutani S. Hares J. M. Murbach M. Green E. W.. (2014a). Genetic analysis of circadian responses to low frequency electromagnetic fields in *Drosophila melanogaster*. PLoS Genet. 10:e1004804. 10.1371/journal.pgen.1004804 25473952 PMC4256086

[B13] Fedele G. Green E. W. Rosato E. Kyriacou C. P. (2014b). An electromagnetic field disrupts negative geotaxis in *Drosophila* via a CRY-dependent pathway. Nat. Commun. 5:4391. 10.1038/ncomms5391 25019586 PMC4104433

[B14] Fogle K. J. Baik L. S. Houl J. H. Tran T. T. Roberts L. Dahm N. A.. (2015). CRYPTOCHROME-mediated phototransduction by modulation of the potassium ion channel β-subunit redox sensor. Proc. Natl. Acad. Sci. U S A 112, 2245–2250. 10.1073/pnas.1416586112 25646452 PMC4343116

[B15] Fogle K. J. Parson K. G. Dahm N. A. Holmes T. C. (2011). CRYPTOCHROME is a blue-light sensor that regulates neuronal firing rate. Science 331, 1409–1413. 10.1126/science.1199702 21385718 PMC4418525

[B16] Fouquet W. Owald D. Wichmann C. Mertel S. Depner H. Dyba M.. (2009). Maturation of active zone assembly by *Drosophila* Bruchpilot. J. Cell Biol. 186, 129–145. 10.1083/jcb.200812150 19596851 PMC2712991

[B17] Gegear R. J. Casselman A. Waddell S. Reppert S. M. (2008). Cryptochrome mediates light-dependent magnetosensitivity in *Drosophila*. Nature 454, 1014–1018. 10.1038/nature07183 18641630 PMC2559964

[B101] Golemis E. A. Brent R. (1997). “Searching for interacting proteins with the two-hybrid system, III,” in The Yeast Two-Hybrid System, eds Bartel P. L. Fields S. (New York, NY: Oxford University Press), 43–72.

[B18] Górska-Andrzejak J. Makuch R. Stefan J. Görlich A. Semik D. Pyza E. (2013). Circadian expression of the presynaptic active zone protein bruchpilot in the lamina of *Drosophila melanogaster*. Dev. Neurobiol. 73, 14–26. 10.1002/dneu.22032 22589214

[B19] Heisenberg M. (1971). Separation of receptor and lamina potentials in the electroretinogram of normal and mutant *Drosophila*. J. Exp. Biol. 55, 85–100. Available online at: www.ncbi.nlm.nih.gov/ pubmed/5001616. 5001616 10.1242/jeb.55.1.85

[B102] Heisenberg M. Wolf R. (1984). Vision in Drosophila: Genetics of Microbehavior. Berlin, New York, NY: Springer-Verlag.

[B20] Hida Y. Ohtsuka T. (2010). CAST and ELKS proteins: Structural and functional determinants of the presynaptic active zone. J. Biochem. 148, 131–137. 10.1093/jb/mvq065 20581014

[B21] Hunter-Ensor M. Ousley A. Sehgal A. (1996). Regulation of the *Drosophila* protein timeless suggests a mechanism for resetting the circadian clock by light. Cell 84, 677–685. 10.1016/s0092-8674(00)81046-6 8625406

[B22] Ishikawa T. Matsumoto A. Kato T. Togashi S. Ryo H. Ikenaga M.. (1999). DCRY is a *Drosophila* photoreceptor protein implicated in light entrainment of circadian rhythm. Genes Cells 4, 57–65. 10.1046/j.1365-2443.1999.00237.x 10231393

[B23] Kittel R. J. Hallermann S. Thomsen S. Wichmann C. Sigrist S. J. Heckmann M. (2006). Active zone assembly and synaptic release. Biochem. Soc. Trans. 34, 939–941. 10.1042/BST0340939 17052232

[B24] Klarsfeld A. Malpel S. Michard-Vanhée C. Picot M. Chélot E. Rouyer F. (2004). Novel features of cryptochrome-mediated photoreception in the brain circadian clock of *Drosophila*. J. Neurosci. 24, 1468–1477. 10.1523/JNEUROSCI.3661-03.2004 14960620 PMC6730330

[B25] Kula E. Pyza E. (2007). Effects of locomotor stimulation and protein synthesis inhibition on circadian rhythms in size changes of L1 and L2 interneurons in the fly’s visual system. Dev. Neurobiol. 67, 1433–1442. 10.1002/dneu.20518 17497696

[B26] Matkovic T. Siebert M. Knoche E. Depner H. Mertel S. Owald D.. (2013). The bruchpilot cytomatrix determines the size of the readily releasable pool of synaptic vesicles. J. Cell Biol. 202, 667–683. 10.1083/jcb.201301072 23960145 PMC3747298

[B27] Mazzotta G. Rossi A. Leonardi E. Mason M. Bertolucci C. Caccin L.. (2013). Fly cryptochrome and the visual system. Proc. Natl. Acad. Sci. U S A 110, 6163–6168. 10.1073/pnas.1212317110 23536301 PMC3625353

[B28] Mazzotta G. M. Costa R. (2016). Circadian control of visual plasticity in arthropods. Ethol. Ecol. Evol. 28, 1–19. 10.1080/03949370.2015.1064037

[B29] Meinertzhagen I. A. O’Neil S. D. (1991). Synaptic organization of columnar elements in the lamina of the wild type in *Drosophila melanogaster*. J. Comp. Neurol. 305, 232–263. 10.1002/cne.903050206 1902848

[B30] Naidoo N. Song W. Hunter-Ensor M. Sehgal A. (1999). A role for the proteasome in the light response of the timeless clock protein. Science 285, 1737–1741. 10.1126/science.285.5434.1737 10481010

[B31] Nilsson D. E. Ro A. I. (1994). Did neural pooling for night vision lead to the evolution of neural superposition eyes? J. Comp. Physiol. A 175, 289–302. 10.1007/BF00192988

[B32] Ozturk N. Selby C. P. Annayev Y. Zhong D. Sancar A. (2011). Reaction mechanism of *Drosophila* cryptochrome. Proc. Natl. Acad. Sci. U S A 108, 516–521. 10.1073/pnas.1017093108 21187431 PMC3021015

[B33] Peschel N. Chen K. F. Szabo G. Stanewsky R. (2009). Light-dependent interactions between the *Drosophila* circadian clock factors cryptochrome, jetlag and timeless. Curr. Biol. 19, 241–247. 10.1016/j.cub.2008.12.042 19185492

[B34] Picot M. Cusumano P. Klarsfeld A. Ueda R. Rouyer F. (2007). Light activates output from evening neurons and inhibits output from morning neurons in the *Drosophila* circadian clock. PLoS Biol. 5:e315. 10.1371/journal.pbio.0050315 18044989 PMC2229858

[B35] Prokop A. Meinertzhagen I. A. (2006). Development and structure of synaptic contacts in *Drosophila*. Semin. Cell Dev. Biol. 17, 20–30. 10.1016/j.semcdb.2005.11.010 16384719

[B36] Pyza E. Górska-Andrzejak J. (2004). Involvement of glial cells in rhythmic size changes in neurons of the housefly’s visual system. J. Neurobiol. 59, 205–215. 10.1002/neu.10307 15085538

[B37] Pyza E. Meinertzhagen I. A. (1997). Circadian rhythms in screening pigment and invaginating organelles in photoreceptor terminals of the housefly’s first optic neuropile. J. Neurobiol. 32, 517–529. 10.1002/(sici)1097-4695(199705)32:5<517::aid-neu6>3.3.co;2-4 9110262

[B38] Pyza E. Meinertzhagen I. A. (1999). Daily rhythmic changes of cell size and shape in the first optic neuropil in *Drosophila melanogaster*. J. Neurobiol. 40, 77–88. 10.1002/(sici)1097-4695(199907)40:1<77::aid-neu7>3.0.co;2-0 10398073

[B39] Ritz T. Yoshii T. Helfrich-Foerster C. Ahmad M. (2010). Cryptochrome: a photoreceptor with the properties of a magnetoreceptor? Commun. Integr. Biol. 3, 24–27. 20539777 10.4161/cib.3.1.9865 PMC2881235

[B40] Rosato E. Codd V. Mazzotta G. Piccin A. Zordan M. Costa R.. (2001). Light-dependent interaction between *Drosophila* CRY and the clock protein PER mediated by the carboxy terminus of CRY. Curr. Biol. 11, 909–917. 10.1016/s0960-9822(01)00259-7 11448767

[B41] Stanewsky R. Kaneko M. Emery P. Beretta B. Wager-Smith K. Kay S. A.. (1998). The cryb mutation identifies cryptochrome as a circadian photoreceptor in *Drosophila*. Cell 95, 681–692. 10.1016/s0092-8674(00)81638-4 9845370

[B42] Wagh D. A. Rasse T. M. Asan E. Hofbauer A. Schwenkert I. Dürrbeck H.. (2006). Bruchpilot, a protein with homology to ELKS/CAST, is required for structural integrity and function of synaptic active zones in *Drosophila*. Neuron 49, 833–844. 10.1016/j.neuron.2006.02.008 16543132

[B43] Wichmann C. Sigrist S. J. (2010). The active zone T-bar—a plasticity module? J. Neurogenet. 24, 133–145. 10.3109/01677063.2010.489626 20553221

[B44] Woźnicka O. Görlich A. Sigrist S. Pyza E. (2015). BRP-170 and BRP190 isoforms of bruchpilot protein differentially contribute to the frequency of synapses and synaptic circadian plasticity in the visual system of *Drosophila*. Front. Cell. Neurosci. 9:238. 10.3389/fncel.2015.00238 26175667 PMC4485229

