# Supplementary material for: Cryptochrome Is a Regulator of Synaptic Plasticity in the Visual System of Drosophila melanogaster
Source: Front Mol Neurosci. 2017 May 30;10:165. doi: 10.3389/fnmol.2017.00165 (PMC5448152; doi:10.3389/fnmol.2017.00165)
Supplement: Supplementary file 1 [file Presentation_1.pdf]

Supplementary Material

Cryptochrome is a regulator of synaptic plasticity in the visual system of *Drosophila melanogaster*

Damulewicz Milena*, Mazzotta Gabriella Margherita, Sartori Elena, Rosato Ezio, Costa Rodolfo, Pyza Elzbieta

*** Correspondence:**

milena.damulewicz@uj.edu.pl

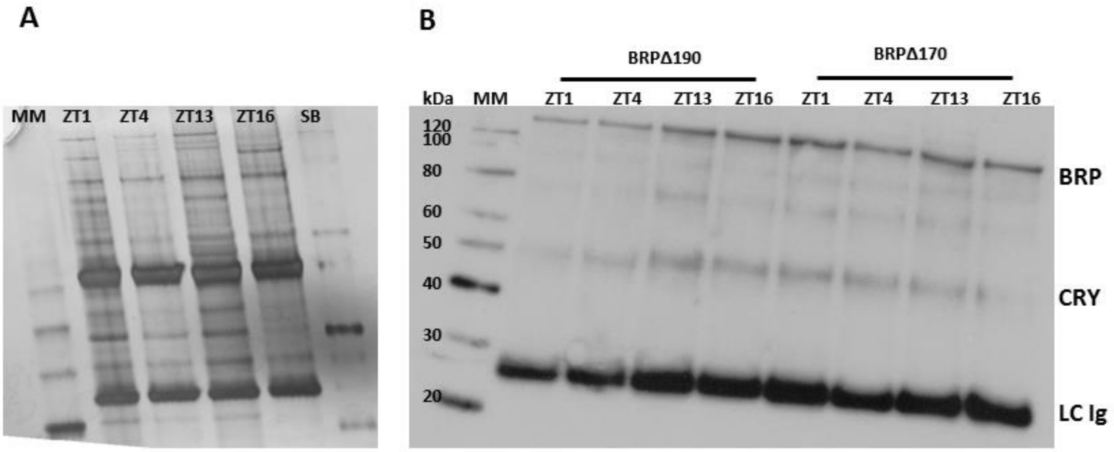

Fig.S1 **BRP forms multiprotein complexes *in vivo***. BRP was precipitated from whole head protein extracts at four time points: ZT1, ZT4, ZT13, ZT16 and then incubated with nc82 antibodies bound onto magnetic beads (Dynabeads). (**A**) Co-immunoprecipitating (coIP) proteins from Canton S flies were resolved by PAGE and then visualized by silver staining on gel. The figure shows that dozens of different proteins may bind to BRP. MM - MagicMark marker, SB- SeaBlue marker. (**B**) coIP proteins from mutants *brp* $^{\Delta 190}$ and *brp*$^{\Delta 170}$ were analyzed by Western blot and then probed with α-BRP and α–CRY primary antibodies. The blot shows the 170kDa and 190kDa BRP isoforms, respectively and a weak signal for the coIP CRY. We can detect a strong cross-hybridization signal from the light chain Immunoglobulin (LC Ig), whereas the secondary antibodies used in this reaction did not recognize the heavy chain Ig. MM- Magic Mark marker.
